# A check list of non-lichenised fungi occurring on *Fagus crenata*, a tree endemic to Japan

**DOI:** 10.1080/21501203.2017.1363092

**Published:** 2017-08-09

**Authors:** Tsuyoshi Hosoya, Kentaro Hosaka, Kyong-Ok Nam

**Affiliations:** Department of Botany, National Museum of Nature and Science, Tsukuba, Japan

**Keywords:** Barcoding, biodiversity, conservation biology, fungal inventory, mycobiota

## Abstract

Non-lichenised fungi from *Fagus crenata*, an endemic and major temperate tree species, were enumerated based on three approaches: fungarium specimens at the National Museum of Nature and Science; isolates obtained mainly from leaves and roots, and their molecular identification by barcoding region; and literature. In total, 209, 49, and 232 taxa were recognised from the fungarium specimens, isolates, and literature, respectively. Only three taxa were commonly observed using all three approaches. Moreover, the results demonstrate the diversity of fungi occurring on a single host plant species, and provide the basis for comparisons between fungi from *Fagus* spp. in other regions of the world.

## Introduction

The genus *Fagus* comprises 10 species and is distributed only in the Northern Hemisphere, with disjunct distribution in Europe, North America, and Asia. In Japan, *Fagus crenata* Blume (Japanese beech) is one of the major deciduous arboreal vegetation in the temperate zone. Some other *Fagus* species are also dominant in Europe (*F. sylvatica*) and America (*F. americana*). Owing to their importance in vegetation, significant numbers of mycological studies have been conducted in *Fagus* forests (Carré 1964; Hogg 1966; Hogg and Hudson 1966; Yamashita et al. 2010; Unterseher et al. 2016).

*Fagus crenata* is an endemic tree species in Japan, forming climax forests. Because of its dominance in Japanese flora and owing to interest in phylogeography and conservation biology, a number of studies have been conducted on *F. crenata* (Kurata 1964; Horikawa 1972; Tsukada 1982; Murai et al. 1991; Fujii et al. 2002; Terazawa and Koyama 2008).

The present study is a part of a larger research project, aiming for evolutionary and phylogeographic studies of the symbiotic ecosystem of *Fagus* species in addition to other research projects. A number of biological interactions between fungi and *F. crenata* (i.e. parasitism, symbiosis, and saprophytism) are known, and a number of studies concerning decomposition and microbiome have been carried out (Osono 2002; Fukasawa et al. 2009; Yamashita et al. 2010; Tateno et al. 2015). Despite the wide range of diversity, records of the occurrence of fungal taxa on *F. crenata* have not been cumulated in an internationally accepted standard format (e.g. Darwin Core) that allows for future data recycling.

For research on fungal biodiversity, determining the fungal diversity on a certain host is a fundamental issue. Such accumulated data can be used to study the distribution, interaction between fungi and plants, evaluation of the coverage of fungal biodiversity, plant protection, and other environmental analyses. In the present paper, we attempted to cumulate the mycobiota of *F. crenata*, hitherto known from the available literature, fungarium specimens, and newly obtained isolates with their molecular-based identification to present a checklist for future studies.

## Materials and methods

Three data sources were investigated: (1) fungarium specimens, (2) endophytic isolates collected from *F. crenata*, and (3) literature. The data obtained from each data set were cumulated in tables. Whenever possible, the field names of the database followed the Darwin Core terms (http://rs.tdwg.org/dwc/) to facilitate compatibility among the data sources. Because significant numbers of taxon names in the obtained data set were out-of-date, the current usage of names was checked using the Index Fungorum (http://www.indexfungorum.org/) to adopt the generic and specific taxonomy in Species Fungorum (http://www.speciesfungorum.org/). Both the names on the original data source and the current names are shown in the data sets (Supplementary Tables 1–4). Phylum rank taxonomy of anamorphic fungi followed the Index Fungorum.

### Fungarium specimens

Specimens housed in the fungarium at the National Museum of Nature and Science (TNS) with *F. crenata* as their host were surveyed. TNS holds the largest mycological collection in Japan covering a wide range of fungi from various substrates and habitats. The data based on field expeditions carried out in some *F. crenata* forests in Japan (Obora, Ueda-shi, Nagano Prefecture [36.503806, 138.328917, 1310 m elev.]) and Tsukuba University Sugadaira Montane Research Center (SMRC, Ueda-shi, Nagano Pref. [36.524697, 138.347102, 1360 m elev.]) between 2011 and 2015 were also incorporated.

Specimens showing physical contact with *F. crenata* by the description on their label were selected. Although some specimens strongly suggested a relationship with *F. crenata* (e.g. collected on the ground in *F. crenata* forest; collected on decaying wood in *F. crenata* forest, etc.), they were discarded because they do not show a physical contact. However, some possible mycorrhizal members were retained, because their association with *F. crenata* was described on the label. Since the substrate or the part of the plant where the fungus occurred were variously described on the specimen labels, they were further categorised with respect to bark, branch, cupule, fruit, leaf, stump, trunk, and wood. The data fields for specimens with uncertain indication for such information (e.g. “on *F. crenata*”) were left blank. When the coordinates of collection sites were not recorded, they were calculated using the “CSV address matching service,” provided by the Center for Spatial Information Science at the University of Tokyo (http://newspat.csis.u-tokyo.ac.jp/geocode-cgi/geocode.cgi?action=start). This service allowed us to calculate the decimal degrees of latitude and longitude based on the provided site names in Japanese. Owing to insufficient information for collection site, this calculation was not carried out for some specimens.

### Endophytic isolates

Endophytic isolates were mostly obtained from fresh leaves and roots, but also from fresh cupules. For leaves and roots, materials from Obora and SMRC were used. For fresh cupules, materials from Chichibu, Tokyo Forest, Tokyo University (35.9428, 138.7980, 1200 m elev.), and Naruko-onsen, Osaki-shi, Miyagi Pref. (38.7304, 140.7679, 120 m elev.) were used. Leaves and roots of *F. crenata* were surface sterilised for 1 min with 70% ethanol followed by 2 min with 1% HClO. After overnight desiccation on filter paper, several 1.0 cm × 1.0 cm pieces from leaves, and 1.0 cm pieces from roots were dissected using a sterile scalpel. The plant pieces were inoculated onto a cornmeal agar (Nissui, Tokyo) plate and were incubated at 17°C. Approximately 2–10 days after inoculation, hyphal tips from the plant substrates were cut and transferred to a potato dextrose agar (PDA, Nissui) plate and allowed to grow for 7–10 days. After comparison of the macro-morphology (e.g. colour, sizes, texture) of colonies from the same plant tissues, only isolates of unique phenotypes were selected within the lot and transferred to PDA slants. For cupules, a longer surface sterilisation procedure was applied (1.5 min with 70% ethanol and 1–5 min with 1% HClO), and small pieces of cupule lobes (5 mm square) were dissected; the remaining procedure was followed as described above.

The resulting isolates were cultivated in 2 ml of 2% malt extract broth for 2 weeks, and the mycelia were harvested and lyophilised. About 50 mg of mycelium was mechanically lysed with a Qiagen TissueLyser (Qiagen, Venlo, The Netherlands) by using ceramic beads, and DNA was extracted by incubation in CTAB buffer (2% CTAB, 100 mM Tris pH 8.0, 20 mM EDTA, 1.4 M NaCl) at 65°C for 1 h. Proteins were removed using a mixture of chloroform:isoamylalcohol (24:1). The materials were further puriﬁed using 6 M sodium iodine buffer with glass milk, washed with ethanol/buffer solution, and ﬁnally eluted in 100 μl of Tris-ethylenediaminetetraacetic acid buffer. The extracted DNA samples were deposited at the Centre for Molecular Biodiversity Research at the National Museum of Nature and Science, and are available for collaborative research upon request.

To amplify the internal transcribed spacers (ITS1 and ITS2) and 5.8S ribosomal region (ITS-5.8S region) used for fungal barcoding, the primer pairs ITS1 or ITS1F and ITS4 (White et al. 1990) were used. The procedure used for polymerase chain reaction (PCR) and sequencing was previously described by Hosoya et al. (2010). The sequencing reaction was carried out using a BigDye terminator cycle sequencing kit (Applied Biosystems, Foster city, CA) using the same primers as for those used PCR amplification. The products were precipitated with ethanol for purification. Sequencing was conducted using an ABI 3130xl Genetic Analyzer (Applied Biosystems).

The resulting barcoding sequences were used for molecular identification using the software Claident ver. 0.2.2016.07.05 (Tanabe and Toju 2013), which seeks sequences in the GenBank database with the highest similarity at a given threshold and returns the most probable identification at the rank of species (or intraspecies) or higher levels (genus or above), if no significant hits are found at the species level. The similarity threshold was set to 97% (sequences with more than 97% similarity were considered conspecific).

### Literature survey

Fungi occurring in Japan have been compiled in a series of checklists updated irregularly by several authors (Shirai 1905; Shirai and Miyake 1917; Shirai and Hara 1927; Hara 1954). The most recent one is by Katumoto (2009) which includes ~12,000 hitherto published Japanese mycobiota until the year 2007 with the host information when available. Our primary survey on fungi occurring on *F. crenata* was based on the list by Katumoto (2009) and other primary sources that indicate the occurrence of fungi on *F. crenata*.

## Results

### Fungarium specimens

In total, 770 specimens occurring on *F. crenata* were found in the fungarium. The data set included 209 species distributed in 133 genera (Supplementary Table 1). However, after the reconciliation of taxon names using the Species Fungorum, the number of names was reduced to 190 species in 130 genera. All the specimens belonged to Ascomycota or Basidiomycota, and the majority of them had macro-morphological structures (e.g. fruit bodies) easily recognisable by naked eyes.

### Endophytic isolates

In total, 364 isolates were obtained and sequenced, and a total of 353 sequences (including duplication) were obtained and examined using Claident. The software provided identification at the species level for 273 isolates. Isolates with the identification “cf.” were dropped from the list. As a result, 254 isolates were identified at the species level (Supplementary Table 2). After adopting the current taxon names, a total of 49 species distributed across 40 genera were enumerated. Most belonged to Ascomycota, but some Basidiomycota (*Amanita tenuifolia, Flammulina velutipes, Schizophyllum commune, Thanatephorus cucumeris, Trametes versicolor*, and *Trichaptum abietinum*), and Mucoromycota (*Syncephalastrum racemosum* and *Umbelopsis dimorpha*) were also recorded (Supplementary Table 2).

### Literature survey

In Katumoto (2009), more than 400 records for 298 taxa (including recurring taxa appearing in multiple pages) were found, but only 133 records were accompanied with a direct indication of *F. crenata* as a host in the primary source (the rest were synonyms of these fungi). In addition, 156 additional records were incorporated into the database extracted from 12 literature sources by further literature survey (Supplementary Table 3). By adopting the current usage of taxon names in the Species Fungorum, the names were rearranged to 232 taxa. One fungus, *Gloeophyllum betulina* in Ueyama (1966), was not found in the Index Fungorum, and was excluded from further analyses.

### Biodiversity of the obtained taxa

The combined data set covered 399 taxa distributed in 260 genera excluding the overlapping taxa (Supplementary Table 4). The majority of the species belonged to Basidiomycota (51.1%), followed by Ascomycota (46.1%), and Mucoromycota (2.8%). No zoosporic fungi were enumerated. The species that appeared in all the three approaches (fungarium specimens, isolates, and literature) were *Dasyscyphella longistipitata, Trametes versicolor*, and *Schizophyllum commune*. The first has been only reported from *F. crenata*, whereas the other two are known from a wide range of host plants.

## Discussion

The present study provides the first cumulated list of species occurring on *F. crenata*, and allows further comparison with other hosts including other *Fagus* species across the world. In the preliminary comparison with the mycobiota on *F. sylvatica* based on the data set provided by Carré (1964), Hogg (1966), Hogg and Hudson (1966), and Unterseher et al. (2016), only a few overlapping species were recognised (e.g. *Acrodontium crateriforme, Epicoccum nigrum, Lachnum virgineum, Schizophyllum commune*, and *Trametes versicolor*). Because the host range of many fungi are limited at the generic level of the plant, the number of overlapping species were lower than expected, but this may be due to the effect of environmental conditions or the different detection methods (Unterseher et al. 2016).

The present study has several limitations. First, we dropped a majority of ectomycorrhizal mushrooms because of the lack of physical evidence of contact with *F. crenata*. Due to their limited ability to grow on artificial media, these fungi are very difficult to detect by isolation. Molecular detection would therefore be used as a complementary method. Second, we followed the Species Fungorum to reconcile taxon names (generic and specific ranks), but the given taxonomic names were not always agreeable. For instance, *Brunnipila fuscescens* is synonymised as *Lachnum fuscescens* in Species Fungorum, but based on our molecular phylogenetic evidence, we do not think that it is an appropriate treatment (Hosoya et al. 2010). However, to discuss the biodiversity in the same framework, we need to standardise name usage under a certain system.

Fröhlich and Hyde (1999) reported 189 species plus 53 unnamed “morphospecies” of fungi from six palms, and suggested the requirement of revision upwards for the estimated ratio of host-specific fungi to the host. Our study provides a higher number of fungi recovered from a single host, and suggests an even more upward revision for the fungi:host ratio. The present study also provides fundamental data for such studies in the future.
